# The computer-assisted interview In My Shoes – a successful method to capture children’s perspectives

**DOI:** 10.1093/eurpub/ckac131.246

**Published:** 2022-10-25

**Authors:** K Fängström, A Dahlberg, A Sarkadi

**Affiliations:** CHAP, Public Health and Caring Sciences, Uppsala University, Uppsala, Sweden

## Abstract

**Background:**

Child mental health problems are considered the second highest cause of burden of disease in Europe and the Americas. Children’s own opinions and experiences are pivotal in addressing these problems. However, including young children as active informants in health research and practice not only requires a well-trained and highly qualified workforce, but also valid methods that enhance and support children’s self-expression. The aim was to investigate preschool aged children’s experiences in two health and welfare contexts using the interactive computer-assisted interview In My Shoes (IMS).

**Methods:**

Interviews were conducted using IMS in three studies encompassing 43 children aged 3-6 years old. The setting for the first and second study was Child Health Centres and the third setting was families entering the Triple P group parenting programme. Qualitative content analysis was performed.

**Results:**

The IMS interview aided preschool aged children to report on the factual, emotional and physical aspects of their experiences within a health care context. In addition, IMS helped young children verbalise unique information on negative interplay within their families, especially experiences of negative parenting including verbal and physical child abuse. The successes with IMS are likely related to the structured and systematic approach, that it is pictorial-based and emotion-focused, as well as the interactive, collaborative and triadic conversation between the child, the interviewer and the computer.

**Conclusions:**

The interactive computer-assisted interview IMS, is a suitable and valid method for aiding young children to provide unique and extensive information about different aspects of their experiences and lives. We urge professionals and researchers to systematically include the young children’s own perspectives to better tailor and evaluate interventions on all levels to improve children’s health and wellbeing.

**Key messages:**

• Young children’s own perspectives on their health and wellbeing are pivotal to better tailor and evaluate interventions.

• The interactive computer-assisted interview In My Shoes is a suitable and valid method to capture children’s voices.

